# Preliminary Results Regarding the Feasibility and Outcomes of MR-Linac Adaptive Stereotactic Body Radiotherapy Combined with Systemic Treatment Among Patients with Pelvic–Abdominal Recurrent or Metastatic Gynecological Malignancies: A Single-Institution Experience

**DOI:** 10.3390/cancers18071112

**Published:** 2026-03-30

**Authors:** Xi Yang, Shuang Zhao, Zexuan Liu, Lu Zhang, Duan Yang, Shuangzheng Jia, Jusheng An, Manni Huang

**Affiliations:** Department of Gynecological Oncology, National Cancer Center/National Clinical Research Center for Cancer/Cancer Hospital, Chinese Academy of Medical Sciences and Peking Union Medical College, Beijing 100021, China; yangxi_pumc@outlook.com (X.Y.); zs18846149423@163.com (S.Z.); zexuanliu95@163.com (Z.L.); happyzl9003@163.com (L.Z.); yangduan1213@163.com (D.Y.); jiashuangzheng@cicams.ac.cn (S.J.)

**Keywords:** image-guided adaptive stereotactic body radiotherapy, gynecological tumors, MR-Linac, combined therapy

## Abstract

Objective: Inadequate radiation delivery to recurrent pelvic and abdominal tumors is frequently attributable to the dose limitations of surrounding normal structures, particularly the intestines. Radiotherapy guided by magnetic resonance imaging (MRI) significantly enhances the accuracy of soft-tissue delineation. The purposes of this study were to demonstrate the feasibility and effectiveness of MR-Linac Adaptive stereotactic body radiotherapy in patients with pelvic–abdominal recurrent or metastatic gynecological malignancies with or without systemic therapies. Methods: Patients with pelvic–abdominal recurrent or metastatic gynecological malignancies are eligible for MR-Linac Adaptive stereotactic body radiotherapy. Systemic therapies, including chemotherapy, immunotherapy, and targeted therapy, are considered acceptable treatment options. The safety, tolerability, and efficacy of MR-Linac Adaptive stereotactic body radiotherapy were assessed. Results: Between October 2019 and May 2025, 15 patients were subjected to MR-Linac Adaptive stereotactic body radiotherapy. With a median follow-up period of 4.67 months (range, 0.73–20.10 months), the 6-month overall survival (OS), progression-free survival (PFS), and local control (LC) rates were 93.3%, 66.0%, and 92.3%, respectively. The 12-month OS, PFS, and LC rates were 83.8%, 37.7%, and 70.5%, respectively. The best objective response rate (ORR = CR + PR) for the irradiated lesions was 73.3% (11/15 patients). MR-Linac Adaptive stereotactic body radiotherapy led to objective responses in 73.3% (11/15) of the patients. As of the data cutoff (28 May 2025), one patient experienced dose-limiting toxicity (an enteric fistula). Another patient developed grade 4 thrombocytopenia during treatment; it was considered chemotherapy-induced. Conclusions: These findings suggest that MR-Linac Adaptive stereotactic body radiotherapy is relatively effective and safe and can be an important treatment option for patients with pelvic–abdominal recurrent or metastatic gynecological malignancies. MR-Linac Adaptive stereotactic body radiotherapy exhibited acceptable tolerability, promising efficacy, and a favorable local control rate with regard to heavily pretreated advanced solid tumors.

## Simple Summary

Patients with recurrent or metastatic gynecological cancers in the abdomen or pelvis often have limited treatment options due to prior therapies and the proximity of tumors to sensitive organs like the bowel. This study investigated whether a new precise radiotherapy technique—MR-Linac adaptive stereotactic body radiotherapy (SBRT)—combined with systemic treatments could be a safe and effective option for these patients. We treated 15 patients and found that this approach was feasible, with treatment successfully adapted daily based on real-time MRI images to account for organ movement. The therapy resulted in a high tumor response rate (73.3%), with only one case of severe late toxicity (an enteric fistula). Our findings suggest that MR-Linac adaptive SBRT combined with systemic therapy represents a valuable, well-tolerated treatment strategy for this challenging patient population, warranting further investigation in larger studies.

## 1. Introduction

Currently, the standard treatment guidelines for recurrent metastatic cervical cancer recommend first-line chemotherapy combined with immunotherapy [1,2]. However, the overall therapeutic benefits remain limited, especially for patients with large local masses accompanied by multiple systemic metastases. Combining systemic therapy with local tumor stereotactic body radiation therapy (SBRT) may offer further benefits to these patients [3], while its safety profile continues to be evaluated in ongoing studies [4].

Recurrence within the pelvic or abdominal cavity is common for patients with gynecological malignancies. For these patients, local radiotherapy is often limited due to prior treatments, such as multiple prior surgeries or radiotherapy, or the increased risk of fistulae associated with bevacizumab [1,5]. The proximity of tumors to radiosensitive organs at risk (OARs), such as the small bowel, colon, bladder, and spinal cord, and the consequential risk of severe toxicity pose major challenges.

Conventional external beam radiotherapy (EBRT), despite being a cornerstone of treatment, is constrained by the tolerance doses of surrounding normal tissues [6]. This frequently results in sub-therapeutic radiation doses being delivered to the target volume, compromising local control (LC). Stereotactic body radiotherapy (SBRT), or stereotactic ablative radiotherapy (SABR), has emerged as a potent technique, delivering highly conformal, ablative doses in few fractions [7]. SBRT’s efficacy hinges on leveraging extreme precision to maximize tumor dose while minimizing exposure to adjacent structures. However, in the mobile and deformable environment of the abdomen and pelvis, interfractional and intrafractional anatomical changes—such as bowel peristalsis, bladder filling, and tumor regression—can lead to targeting inaccuracies and potential geographical misses or OAR overdosing.

The integration of magnetic resonance imaging with a linear accelerator (MR-Linac) has ushered in a new era of adaptive radiotherapy. This technology provides superior soft-tissue contrast relative to conventional cone-beam CT (CBCT), enabling exquisite visualization of both target volumes and OARs during treatment. More importantly, it facilitates a “see, treat, and adapt” workflow. Online adaptive replanning based on daily MRI allows for compensation of anatomical variations, potentially permitting an increase in the dose delivered to the tumor and/or further sparing OARs [8,9,10]. This capacity is particularly crucial in the re-irradiation setting, where the tolerance of previously irradiated tissues is significantly reduced.

While the technical feasibility of MR-Linac adaptive SBRT has been established, robust clinical data on its application specifically for pelvic–abdominal recurrent/metastatic gynecological cancers remain scarce. Furthermore, the optimal integration of this focal ablative therapy with contemporary systemic agents—including chemotherapy, immunotherapy (e.g., immune checkpoint inhibitors), and targeted therapies—is an area of active exploration [11]. This combination holds the promise of producing synergistic effects, addressing both local and disseminated disease. Nevertheless, attention must also be paid to the safety of this combination therapy.

Therefore, the primary objectives of this retrospective study were to evaluate the feasibility, safety, and preliminary efficacy of MR-Linac adaptive SBRT combined with systemic treatment for patients with recurrent or metastatic gynecological malignancies in the challenging pelvic–abdominal region.

## 2. Materials and Methods

### 2.1. Study Design and Patient Population

A single-institution, retrospective observational study was conducted between October 2019 and May 2025. The study was approved by the Ethics Committee of National Cancer Center/Cancer Hospital, Chinese Academy of Medical Sciences and Peking Union Medical College (25/221-5167).

The inclusion criteria were (1) histologically confirmed gynecological malignancy (cervical, endometrial, ovarian, vulvar, etc.); (2) radiologically confirmed recurrent or metastatic lesion (s) within the abdominal or pelvic cavity deemed suitable for localized SBRT by a multidisciplinary tumor board; (3) lesion ≤ 5 cm in maximum diameter; (4) an ECOG performance status of 0–2; and (5) a life expectancy > 3 months. The exclusion criteria included (1) uncontrolled metastatic disease requiring urgent systemic therapy; (2) contraindication for MRI; and (3) prior radiotherapy with overlapping fields wherein cumulative OAR constraints could not be met.

### 2.2. Treatment Planning and Delivery

Simulation: All patients underwent a dedicated planning session using the MR-Linac system (Elekta Unity, Elekta AB, Stockholm, Sweden). A thermoplastic immobilization device was used. A high-resolution 3D T2-weighted MRI scan was acquired for contouring.

Contouring: The gross tumor volume (GTV) was delineated on the planning MRI. A 3–5 mm isotropic expansion was used to create the planning target volume (PTV), with adjustments made to respect anatomical boundaries. Critical OARs (small bowel, colon, stomach, kidneys, spinal cord, bladder, and femoral heads) were contoured meticulously.

Planning: A dose of 30–50 Gy delivered in 5–10 fractions was prescribed for the PTV, with the goal of covering ≥95% of the PTV with ≥95% of the prescription dose. The primary dose-limiting OAR was the small bowel/colon, with strict constraints applied (e.g., V35Gy < 0.5 cc, and a maximum point dose < 38 Gy in 5 fractions for re-irradiation cases).

Adaptive Workflow: On each treatment day, a pre-treatment MRI was acquired. The treating radiation oncologist and physicist evaluated each patient’s anatomy daily. If significant deviations (bowel loop intrusion, large tumor shift/regression, etc.) that would compromise PTV coverage or exceed OAR constraints on the original plan were observed, an online adapt-to-shape (ATS) workflow was employed. A new treatment plan was optimized based on the daily anatomy, re-contouring targets and OARs as needed, and the adapted plan was implemented immediately. If a patient’s anatomy was favorable, the original plan was implemented (adapt-to-position, ATP).

Dose Constraints and Planning Prioritization: Dose constraints were derived from established guidelines and adapted for the re-irradiation setting based on international consensus. For primary SBRT cases, constraints were based on the AAPM TG-101 report. For re-irradiation cases, constraints were adapted from the ESTRO-EORTC re-irradiation consensus and the pelvic SABR re-irradiation Delphi consensus. Cumulative dose constraints were applied considering prior dose, time interval, and organ-specific recovery assumptions where evidence exists (e.g., for spinal cord).

In the planning process, we distinguished between “hard” constraints that were typically not exceeded (e.g., small bowel Dmax < 38 Gy in 5 fractions, spinal cord Dmax < 25 Gy in 5 fractions, or cumulative EQD2 < 50 Gy for re-irradiated spinal cord) and “soft” constraints that could be relaxed if clinically justified. When OAR sparing was prioritized, a minimum of 70% of the PTV receiving the prescribed dose was considered acceptable, in accordance with the ESTRO-EORTC consensus recommendation. Constraints were applied flexibly and individualized based on tumor location, proximity to critical OARs, prior treatment history, and patient-specific factors such as life expectancy and symptom burden, consistent with the ESTRO-EORTC guidance that prioritization should be guided by the patient’s risk acceptance and treatment goals.

Cumulative Dose Assessment for Re-irradiated Patients: For all patients with a history of prior radiotherapy, the previous radiotherapy plans were available and reviewed. Due to the lack of deformable registration capabilities and the limitations of rigid fusion for pelvic anatomies, precise dose summation was not feasible. Instead, we applied a conservative worst-case estimation approach to ensure safety. Specifically, we assumed that the maximum dose points from the prior treatment (prior hotspots) and the current Dmax limits were located in the same anatomical region of each organ at risk. Cumulative doses were then estimated by summing these values after conversion to equivalent dose in 2 Gy fractions (EQD2) using organ-specific α/β assumptions (α/β = 3 Gy for late-responding tissues such as bowel, bladder, and rectum). This conservative methodology provides a safety-oriented estimate of cumulative organ-at-risk doses, acknowledging the inherent uncertainties in cumulative dose assessment. The detailed treatment parameters for these individual lesions are summarized in Supplementary Table S1.

### 2.3. Systemic Therapies

Concurrent or sequential systemic therapy was permitted at the discretion of the oncologist. The corresponding therapies included chemotherapy, immunotherapy (e.g., PD-1/PD-L1 inhibitors), and/or targeted therapies. Three patients were administered immune checkpoint inhibitors (pembrolizumab, toripalimab, and tislelizumab) during treatment; eight patients underwent chemotherapy (single-agent cisplatin, paclitaxel plus carboplatin, paclitaxel plus cisplatin, and docetaxel plus cisplatin); and five patients underwent targeted therapies (nimotuzumab and bevacizumab). Administration within ±28 days of the SBRT course was recorded. Systemic Therapy Details by Patient are summarized in Supplementary Table S2.

### 2.4. Outcome Assessment and Follow-Up

Patients were assessed for acute toxicity (≤90 days post-SBRT) and late toxicity (>90 days) using the Common Terminology Criteria for Adverse Events (CTCAE) version 5.0. Clinical and radiological follow-ups (with contrast-enhanced CT or MRI) occurred at 1–3 months post-treatment, followed by every 3–6 months. Tumor response was evaluated according to Response Evaluation Criteria in Solid Tumors (RECIST) v1.1 as a complete response (CR), a partial response (PR), stable disease (SD), or progressive disease (PD). Local control (LC) was defined as the absence of progression within the irradiated PTV. Overall survival (OS) was calculated from the start of SBRT to death via any cause. Progression-free survival (PFS) was defined as ranging from the start of SBRT to disease progression (local or distant) or death.

### 2.5. Statistical Analysis

Descriptive statistics were used for patient characteristics and toxicity. Time-to-event endpoints (OS, PFS, and LC) were analyzed using the Kaplan–Meier method. All analyses were performed using SPSS software (version 26.0, IBM Corp., Armonk, NY, USA).

## 3. Results

### 3.1. Patient and Treatment Characteristics

A total of 15 patients with 18 treated lesions were enrolled. The median age was 57.3 years (range: 35–73). Patient demographics and baseline disease characteristics are detailed in Table 1. The most common primary tumor was cervical cancer (73.3%). Most of the patients (80%) had undergone prior radiotherapy for the region involved.

### 3.2. Treatment Feasibility and Adaption

All planned treatment fractions were successfully delivered using MR-Linac. The online adaptive workflow (ATS) was utilized in 65% of all treatment fractions, primarily due to variations in bowel position and filling. The median time for the entire adaptive session (imaging, re-contouring, re-optimization, pre-treatment check, and delivery) was 45 min.

### 3.3. Efficacy Outcomes

**Figure 1:**
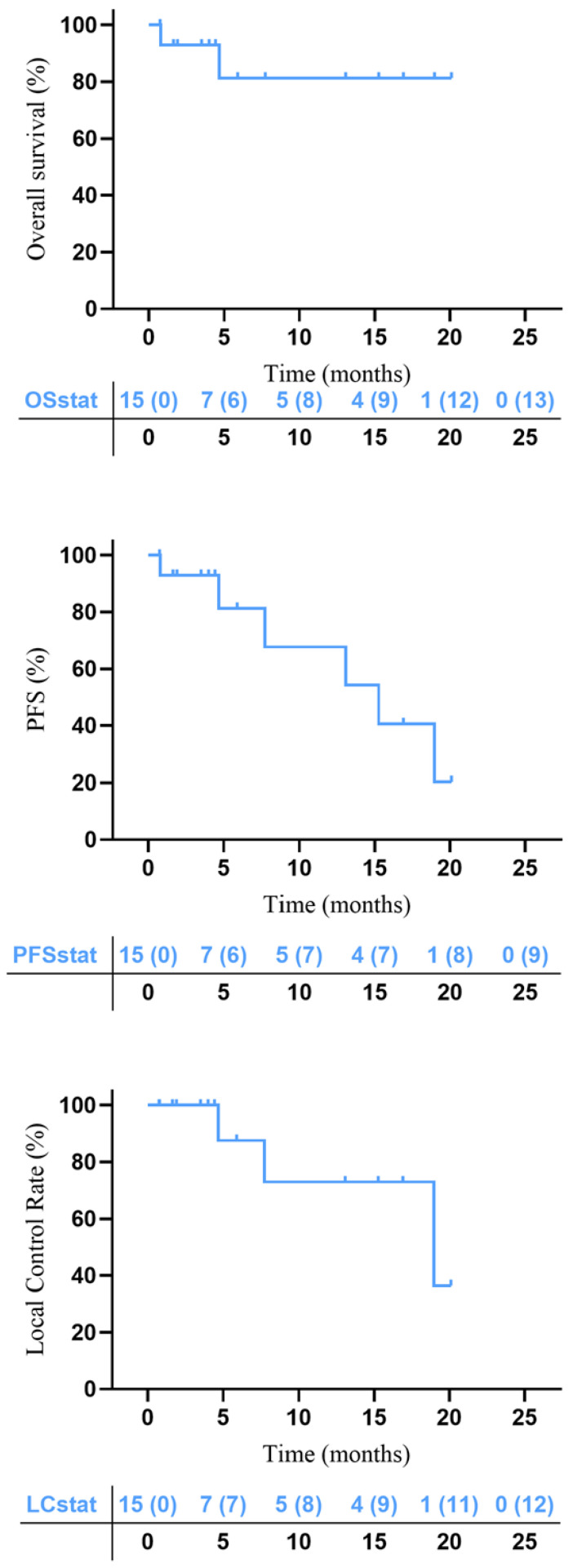
Estimated overall survival, progression-free survival, and local control rate curves.

**Figure 2:**
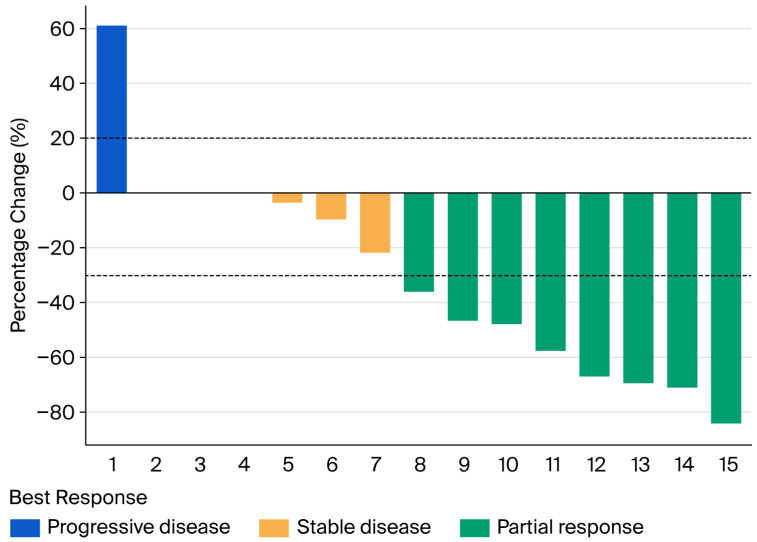
Waterfall plot of best tumor response. The *y*-axis shows the best percentage change from baseline in the sum of target lesion diameters. Each bar represents an individual patient. Colors indicate response category per RECIST v1.1: green = partial response (PR), orange = stable disease (SD), blue = progressive disease (PD). The dashed horizontal line at −30% marks the threshold for partial response; the dotted line at +20% marks the threshold for progressive disease.

With a median follow-up of 4.67 months (range: 0.73–20.10 months), the 6-month overall survival (OS), progression-free survival (PFS), and local control (LC) rates were 93.3%, 66.0%, and 92.3%, respectively. The numbers of patients at risk at 6 months were 14 for OS, 9 for PFS, and 13 for LC. The 12-month OS, PFS, and LC rates were 83.8%, 37.7%, and 70.5%, respectively, with 10, 4, and 7 patients remaining at risk at 12 months. (Figure 1). The best objective response rate (ORR = CR + PR) was 73.3% (11/15 patients). A waterfall plot depicting the best percentage change in patient level is shown in Figure 2. A total of two patients died, with one death attributed to massive tumor hemorrhage and one death resulting from systemic disease progression.

### 3.4. Toxicity Profile

Treatment was generally well-tolerated. Acute toxicities were primarily grade 1–2 fatigue (*n* = 7) and nausea (*n* = 4). One patient (Patient 6) developed CTCAE grade 4 thrombocytopenia during treatment; this development was attributed to concurrent chemotherapy. Regarding late toxicity, one patient developed a grade 3 enteric fistula approximately 8 months post-SBRT. This patient had undergone multiple prior surgeries and radiotherapy for recurrent cervical cancer, and the fistula occurred at the site of a high-dose gradient near the small bowel. No other grade ≥ 3 late toxicities were observed. Detailed patient-level treatment and toxicity data are presented in Table 2.

## 4. Discussion

Numerous studies have demonstrated that SBRT combined with immunotherapy exhibits sensitization mechanisms. For instance, radiotherapy-driven stress signals remodel the tumor immune microenvironment (TIME) through theoretical principles, positively activating the tumor microenvironment and enhancing anti-tumor immune responses [12,13]. Another mechanism is the indirect upregulation of immune checkpoint molecules such as PD-L1 on tumor-cell surfaces through mechanisms like increasing levels of interferon-γ [14,15]. Clinical studies of SBRT combined with immunotherapy for other types of cancer have also provided data confirming the abscopal effect and synergistic benefits of radiotherapy combined with immune checkpoint inhibitor (ICI) therapy [16,17,18].

For patients with persistent, recurrent, or metastatic cervical and endometrial cancer who have undergone prior treatment, traditional chemotherapy and targeted therapies offer limited efficacy. Even in recent years, although immune checkpoint inhibitors such as pembrolizumab have become part of the standard treatment regimen, their monotherapy response rates remain low, and most patients fail to achieve a sustained clinical benefit. The PRIMMO study evaluated the efficacy and safety of pembrolizumab combined with stereotactic body radiation therapy (SBRT) and an immunomodulatory five-drug cocktail in patients with pretreated persistent, recurrent, or metastatic cervical or endometrial carcinoma [3]. The findings indicated that the combination of pembrolizumab, SBRT, and the immunomodulatory cocktail exhibited limited clinical activity in the pretreated advanced cervical and endometrial cancer patients, though it provided durable disease control for a subset of individuals. The regimen demonstrated notable toxicity, highlighting the need for more precise strategies to enhance efficacy and manage toxicities.

Radiation therapy technology is becoming increasingly advanced [19]. Compared to conventional radiotherapy techniques, MR-Linac adaptive radiotherapy can further enhance the localized radiation dose while better protecting surrounding healthy organs [20]. This retrospective study demonstrates that MR-Linac adaptive SBRT is a clinically feasible, safe, and potentially effective treatment modality for patients with recurrent or metastatic gynecological malignancies in the challenging pelvic–abdominal region, a finding that is consistent with most current evidence [21,22,23]. Our findings add evidence supporting the clinical implementation of online adaptive radiotherapy combined with system treatment.

The high rate of online plan adaptation (65% of fractions) underscores the significant interfractional variability in this anatomical region. This adaptability is the cornerstone of MR-Linac’s potential benefit. By accounting for daily changes, we can theoretically maintain tighter treatment margins, which may translate into two key advantages: (1) the ability to safely escalate the dose delivered to the target, potentially improving local control, and (2) reduced irradiation of normal tissues, potentially lowering toxicity. The single case of severe late toxicity (an enteric fistula) occurred in a heavily pre-treated patient with a complex surgical history (the patient had a treatment history consisting of a radical hysterectomy for cervical cancer, postoperative radiotherapy, and pelvic exenteration upon pelvic recurrence, and her current treatment involves re-irradiation within the previously irradiated field), highlighting that even with advanced technology, the risk in re-irradiation scenarios remains non-negligible and calls for extreme caution in planning and patient selection.

The observed 6-month and 12-month LCR and OS are encouraging in this cohort of predominantly pre-irradiated, advanced patients. The high objective response rate (73.3%) indicates a potent ablative effect. Notably, the PFS was low, reflecting the advanced, systemic nature of the disease in this population. This evidence shows that local therapy, no matter how precise, must be integrated into a comprehensive management strategy that addresses disseminated disease. The high rate of systemic therapy in our cohort (60%) reflects this modern multimodal approach, and the favorable safety profile suggests that MR-Linac SBRT can be successfully combined with various agents.

Our study has several limitations. The sample size was small, and the follow-up period was relatively short, especially for assessing late toxicities. The heterogeneity in histology, prior treatments, and concurrent therapies limits broad generalizations. Furthermore, as a single-arm study, we cannot definitively attribute the outcomes solely to the MR-Linac technology as opposed to conventional SBRT. A direct comparative study would be needed to quantify the incremental clinical benefit of the adaptive workflow.

Future research should focus on larger, multi-institutional studies with longer follow-ups to better define efficacy and late-toxicity profiles. Biomarker research is needed to identify the patients most likely to benefit from this resource-intensive therapy. Additionally, the impact of clinically relevant factors such as the platinum-free interval—a well-established prognostic factor in gynecological cancers—should be evaluated in more homogenous patient cohorts. Exploring synergistic combinations with immunotherapy also remains a compelling avenue, as high-dose radiotherapy can modulate the tumor microenvironment and potentially enhance systemic immune responses.

We acknowledge that the radiotherapy planning in this early cohort was largely individualized based on institutional expert consensus rather than a standardized protocol, due to the heterogeneity of lesion locations, prior treatment histories, and concurrent systemic therapies. While this approach reflects real-world clinical decision-making, it limits the reproducibility and generalizability of our findings. These preliminary results should therefore be validated in larger, more standardized cohorts. We hope that our accumulated experience with MR-Linac adaptive SBRT will contribute to the development of more structured treatment protocols in the future.

## 5. Conclusions

MR-Linac adaptive SBRT is a technically feasible and safe treatment option for patients with pelvic–abdominal recurrent or metastatic gynecological malignancies. It yields promising local control and survival outcomes with an acceptable toxicity profile, even in a heavily pre-treated population. Its online adaptive ability is frequently utilized and appears critical for managing anatomical uncertainty in this region. This modality represents a valuable tool in the modern, multidisciplinary management of complex gynecological oncology cases, warranting further investigation in larger prospective studies.

## Figures and Tables

**Table 1:** Demographic and baseline characteristics.

Characteristics	*N* (%)
ECOG performance status	
0	2 (13.3%)
1	13 (86.7%)
Cancer type	
Cervical cancer	11 (73.3%)
Endometrial cancer	2 (13.3%)
Vulvar cancer	1 (6.7%)
Metastatic squamous cell carcinoma of unknown primary	1 (6.7%)
Histopathology	
Squamous cell carcinoma	11 (73.3%)
Adenocarcinoma	2 (13.3%)
Endometrioid carcinoma	2 (13.3%)
PD1/PD-L1 status	
Positive	8 (53.3%)
Negative	1 (6.7%)
Unknown	6 (40%)
Sites of recurrent or metastatic sites	
Abdomen	4 (26.7%)
Pelvic	11 (73.3%)
Pattern of recurrent or metastases	
In-field recurrent	9 (60%)
Out-of-field recurrent	3 (20%)
No history of radiotherapy	3 (20%)

**Table 2:** Treatment details, clinical responses, and adverse events.

	Response of Target Tumor	Vital Status	Pattern of Recurrent or Metastases	ICI *	Chemotherapy *	Targeted Therapy *	Acute Toxicity	Late Toxicity
Patient 1	SD	Dead	In field	-	Yes	-	-	-
Patient 2	PR	Alive	In field	-	-	-	-	-
Patient 3	PD	Alive	Out of field	-	-	-	-	-
Patient 4	PR	Alive	In field	-	-	Yes	-	-
Patient 5	PR	Alive	Out of field	Yes	Yes	Yes	-	-
Patient 6	PR	Alive	No RT history	-	Yes	-	G4 thrombocytopenia	-
Patient 7	SD	Dead	In field	-	Yes	-	-	Enteric fistula
Patient 8	SD	Alive	In field	Yes	Yes	Yes	-	-
Patient 9	PR	Alive	No RT history	-	-	-	-	-
Patient 10	SD	Alive	Out of field	-	-	-	-	-
Patient 11	SD	Alive	In field	-	-	-	-	-
Patient 12	PR	Alive	In field	-	Yes	Yes	-	-
Patient 13	SD	Alive	No RT history	-	Yes	Yes	-	-
Patient 14	PR	Alive	In field	Yes	YES	-	-	-
Patient 15	PR	Alive	In field	-	-	-	-	-

Abbreviations: ICI, immune checkpoint inhibitor; -, not available; PD, progressive disease; PR, partial response; RT, radiotherapy; SD, stable disease. * Application occurred over ±28 days relative to the radiotherapy period.

## Data Availability

The data presented in this study are available from the corresponding authors on request due to ethical and privacy restrictions, as the data are derived from retrospective clinical records and contain potentially identifiable patient information.

## Supplementary Materials

The following supporting information can be downloaded at: https://www.mdpi.com/article/10.3390/cancers18071112/s1, Table S1: Detailed Treatment Parameters for Individual Lesions (*n* = 18); Table S2: Systemic Therapy Details by Patient.
