# Supplementary material for: Preliminary Results Regarding the Feasibility and Outcomes of MR-Linac Adaptive Stereotactic Body Radiotherapy Combined with Systemic Treatment Among Patients with Pelvic–Abdominal Recurrent or Metastatic Gynecological Malignancies: A Single-Institution Experience
Source: Cancers (Basel). 2026 Mar 30;18(7):1112. doi: 10.3390/cancers18071112 (PMC13072319; doi:10.3390/cancers18071112)
Supplement: Supplementary file 1 [file cancers-18-01112-s001.zip › cancers-4169438-supplementary.pdf]

Table S1. Detailed Treatment Parameters for Individual Lesions (n=18)

Lesion No.	Patient ID	Lesion Site	Re-irradiation	Prescription Dose (V95%) / Fractions(EQD2)	ATS/ATP
1	Patient 1	Right iliac vessel LNM	Yes	PGTVnd: 10.5 Gy/3f (11.8 Gy)	ATP
2	Patient 2	Pelvic LNM	Yes	PGTVnd: 9 Gy/3f (9.8 Gy)	ATP
3	Patient 3	Right external iliac/obturator LNM	No	PGTVnd: 60 Gy/12f (75Gy)	ATS (3/9f)
4	Patient 4	Right paraureteral tumor	Yes	PGTV: 8.8 Gy/4f (8.9Gy)	ATP
5	Patient 5	Right psoas muscle medial	No	PGTV: 60 Gy/10f (80Gy)	ATS (2/8f)
6	Patient 6	Retroperitoneal LNM	No	PGTVnd: 70 Gy/14f (87.5Gy)	ATS (3/11f)
7	Patient 7	Right pelvic mass	Yes	PGTV: 15 Gy/3f (18.8Gy)	ATP
8	Patient 8	Pelvic tumor	Yes	PGTV: 57 Gy/15f (65.6Gy)	ATS (3/12f)
9	Patient 9	Retroperitoneal LNM	No	PGTVnd: 35 Gy/5f (49.6Gy)	ATP
10	Patient 10	Peritoneal lesion anterior to bladder	No	PGTV: 30 Gy/5f (40Gy)	ATP
11	Patient 11	Right pelvic wall	Yes	PGTV: 18 Gy/3f (24Gy)	ATP
12	Patient 12	Right parametrial	Yes	PGTV: 40 Gy/8f (50Gy)	ATS (2/6f)
13	Patient 13	Left iliac LNM	No	PGTVnd: 60 Gy/10f (80Gy)	ATP
14	Patient 14	Left iliac LNM	Yes	PGTVnd: 11.7 Gy/3f (13.6Gy)	ATP
15	Patient 15	Retroperitoneal LNM	Yes	PGTVnd: 45.6 Gy/8f (59.7Gy)	ATS (2/6f)
16	Patient 3 (second lesion)	Right iliac LNM (beside the first lesion)	No	PGTVnd: 60 Gy/12f (75Gy)	ATS (3/9f)
17	Patient 6 (second lesion)	Para-aortic LNM (beside the first lesion)	No	PGTVnd: 70 Gy/14f (87.5Gy)	ATS (3/11f)
18	Patient 8 (second lesion)	Pelvic sidewall (beside the first lesion)	Yes	PGTV: 57 Gy/15f (65.6Gy)	ATS (3/12f)

Notes: For patients with multiple lesions treated within the same plan; individual lesion-specific metrics were not available. EQD2 calculated with $\alpha/\beta = 10$ Gy. ATS: Adapt-to-Shape; ATP: Adapt-to-Position. LNM: lymph node metastasis; PGTV: planning gross tumor volume; PGTVnd: planning gross tumor volume for lymph node metastasis.

Table S2. Systemic Therapy Details by Patient

Patient ID	Concurrent Systemic Therapy (during SBRT)	Sequential Systemic Therapy (±28 days)	Anti-VEGF Exposure (Bevacizumab)	Timing of Anti-VEGF Relative to SBRT	Additional information
Patient 1	No	No	No	N/A	Prior concurrent chemoradiotherapy 7 days before SBRT
Patient 2	No	No	No	N/A	Prior concurrent chemoradiotherapy 4 days before SBRT
Patient 3	No	Yes (Paclitaxel liposome + Cisplatin + Tislelizumab)	No	N/A	Pre-SBRT: 2 cycles; Post-SBRT: 4 cycles
Patient 4	No	Yes (Nimotuzumab)	No	N/A	Pre-SBRT: 2 cycles; Post-SBRT: 3 cycles
Patient 5	No	Yes (Pembrolizumab + Bevacizumab + Paclitaxel + Carboplatin)	Yes	28 days before & after SBRT	Pre-SBRT: 2 cycles; Post-SBRT: 4 cycles
Patient 6	Yes (Cisplatin)	No	No	N/A	Concurrent cisplatin x2 cycles
Patient 7	No	Yes (Paclitaxel liposome + Carboplatin)	No	N/A	Post-SBRT: 2 cycles
Patient 8	No	Yes (Cardonilumab + Nimotuzumab)	Yes	>10 months before SBRT	Pre-SBRT: 1 cycles; Post-SBRT: 3 cycles
Patient 9	No	Yes (Megestrol acetate)	No	N/A	Post-SBRT: megestrol acetate maintenance only
Patient 10	No	Yes (Tislelizumab)	No	N/A	Pre-SBRT: 6 cycles; Post-SBRT: 5 cycles
Patient 11	No	Yes (Paclitaxel + Cisplatin)	No	N/A	Pre-SBRT: 5 cycles
Patient 12	Yes	Yes (Paclitaxel liposome + Carboplatin + Tislelizumab)	No	N/A	Pre-SBRT: 3 cycles; Post-SBRT: 5 cycles
Patient 13	No	Yes (Paclitaxel liposome + Carboplatin + Bevacizumab)	Yes	28 days before SBRT	Pre-SBRT: 6 cycles
Patient 14	No	No	No	N/A	Prior concurrent chemoradiotherapy + immunotherapy 4 days before SBRT
Patient 15	No	Yes (Paclitaxel + Cisplatin + Tislelizumab)	No	N/A	Pre-SBRT: 8 cycles

Abbreviations: SBRT, stereotactic body radiotherapy; VEGF, vascular endothelial growth factor; N/A, not applicable.
